# Assessment of optic nerve sheath diameter using ultrasonography in patients with clinical and radiological suspicion of elevated ıntracranial pressure in the ıntensive care unit

**DOI:** 10.1590/1806-9282.20251044

**Published:** 2026-04-20

**Authors:** Aslı Arican Çelik, Ahmet Bindal, Süleyman Sencer Çelik, Pınar Karabacak, Yasemin Görgülü, Vedat Ali Yürekli

**Affiliations:** 1 Suleyman Demirel University, Faculty of Medicine, Department of Anesthesiology and Reanimation – Isparta, Türkiye.; 2 Suleyman Demirel University, Faculty of Medicine, Department of Critical Care – Isparta, Türkiye.; 3 Suleyman Demirel University, Faculty of Medicine, Department of Neurology – Isparta, Türkiye.

**Keywords:** Brain injury, Intracranial pressure, Computed tomography, Optic nerve, Ultrasonography

## Abstract

**OBJECTIVE:**

The aim of this study was to evaluate optic nerve sheath diameter using ultrasonography in patients with clinical and radiological suspicion of elevated intracranial pressure in the intensive care unit. Elevated intracranial pressure is a critical condition resulting from various intracranial pathologies and constitutes a major cause of morbidity and mortality.

**METHODS:**

Several invasive and noninvasive methods are available for detecting elevated intracranial pressure. Although invasive methods are regarded as the gold standard, they are associated with certain disadvantages. Ultrasonographic measurement of optic nerve sheath diameter, a noninvasive technique, has gained prominence owing to its repeatability, bedside applicability, and cost-effectiveness. From June to December 2023, 50 patients with suspected elevated intracranial pressure based on cranial computed tomography findings and clinical presentation, along with 36 healthy volunteers, were included in the study.

**RESULTS:**

Optic nerve sheath diameter was significantly greater in patients (right 6.13±0.50 mm, left 6.13±0.51 mm) compared with controls (right 4.23±0.22 mm, left 4.23±0.21 mm; p<0.001). Moreover, optic nerve sheath diameter was significantly correlated with mortality, as well as with Glasgow Coma Scale, Full Outline of UnResponsiveness, Acute Physiology and Chronic Health Evaluation II, and Sequential Organ Failure Assessment scores. Patients with computed tomography findings such as brain edema and midline shift also exhibited significantly elevated optic nerve sheath diameter values.

**CONCLUSION:**

Ultrasonographic measurement of optic nerve sheath diameter represents a reliable adjunctive modality for identifying patients with radiological signs of elevated intracranial pressure and for prognostic assessment in the intensive care unit. Nevertheless, the lack of invasive intracranial pressure monitoring as the gold standard constitutes a major limitation, and validation against direct intracranial pressure measurements is warranted in future studies. This technique may be particularly useful in hemodynamically unstable patients who cannot be safely transferred for computed tomography imaging.

## INTRODUCTION

The cranium is composed of three main components: brain parenchyma (approximately 80%), cerebrospinal fluid (CSF, approximately 10%), and cerebral blood volume (approximately 10%). Intracranial pressure (ICP) refers to the pressure within the intracranial compartment, which is normally maintained within a relatively narrow range. In pathological conditions, intracranial volume may increase, leading to alterations in ICP^1^. Brain tissue is largely considered incompressible. To maintain stable ICP, a balance must be sustained between arterial inflow, venous outflow, and cerebrospinal fluid production and drainage. This concept is described by the Monro-Kellie hypothesis^2^. When compensatory mechanisms fail, even minor increases in intracranial volume can result in substantial elevations in ICP^2^.

Both invasive and noninvasive methods are employed for measuring ICP. Invasive techniques remain the gold standard but require surgical intervention and carry potential risks such as hemorrhage and infection. Consequently, interest in noninvasive methods has increased in recent years^3^. Noninvasive approaches include computed tomography (CT), cranial magnetic resonance imaging (MRI), transcranial Doppler (TCD), intraocular pressure measurement, venous ophthalmodynamometry, tympanic membrane displacement, and ultrasonographic measurement of the optic nerve sheath diameter (ONSD)^4^.

Certain cranial CT findings are associated with elevated intracranial pressure and play a crucial role in clinical monitoring. Nevertheless, transporting hemodynamically unstable patients to the CT unit and the high radiation exposure remain significant limitations^5^. Ultrasonographic measurement of ONSD has recently emerged as a valuable noninvasive technique due to its ease of learning, bedside applicability, absence of radiation risk, and reproducibility^6^.

The optic nerve sheath is a continuation of the dura mater, with the subarachnoid space located between the sheath and the optic nerve, extending along its course. Consequently, the sheath is influenced by elevated intracranial pressure: as ICP rises, the sheath expands^7^. Therefore, ONSD is considered a reliable indicator of intracranial alterations. Its measurement is easily accessible and can provide valuable prognostic information. Accordingly, this study investigated the relationship between ONSD, cranial CT findings suggestive of elevated ICP, and mortality using various scoring systems.

## METHODS

This study was approved by the Clinical Research Ethics Committee of Süleyman Demirel University (Decision No: 129, June 22, 2023; Project title: Assessment of Optic Nerve Sheath Diameter Using Ultrasonography in Patients with Clinical and Radiological Suspicion of Elevated Intracranial Pressure). The study was conducted in the intensive care unit (ICU) between June and December 2023.

The study included two groups: 50 ICU patients (aged 18–85) with clinical and radiological suspicion of elevated ICP based on cranial CT findings (e.g., edema, midline shift, effaced cisterns) and clinical presentation, and 36 healthy volunteers (aged 18–85) with no history of comorbidities, head trauma, cerebrovascular disease, or cranial surgery. Exclusion criteria comprised age <18 or >85 years, pregnancy, inadequate ocular ultrasonography image quality, globe trauma or periocular open injuries, and periocular infections such as cellulitis.

Control group volunteers underwent ONSD measurements on days 0, 1, 3, and 5, with no additional procedures performed. In the patient group, ONSD measurements were conducted on the same days following ICU admission and were compared with cranial CT findings. Data regarding transfer to the ward or mortality were recorded. Additionally, Glasgow Coma Scale (GCS), Acute Physiology and Chronic Health Evaluation II **(**APACHE II), Sequential Organ Failure Assessment **(**SOFA), and Full Outline of UnResponsiveness **(**FOUR) scores were collected on days 0, 1, 3, and 5.

Measurements were performed using a 7.5–10 MHz linear probe with patients in a supine position and the head elevated at 30°. ONSD was measured 3 mm posterior to the optic disc at its thickest point, in two planes (horizontal and sagittal) for each eye. The mean value was recorded.

### Statistical analysis

Power analysis was performed using GPower 3.1.9.2, and statistical analyses were conducted with SPSS 25.0. Descriptive statistics are presented as means±standard deviations, medians, and interquartile ranges (Q1–Q3) for numerical data, and frequencies (percentages) for categorical variables. Chi-square tests assessed relationships between categorical variables, Pearson correlation analysis was used for numerical variables, and Spearman's Rho was applied when normal distribution was not met. ONSD measurements between two independent groups were compared using Student's t-test. Receiver operating characteristic (ROC) analysis was applied to ONSD values to distinguish the patient group from the control group. A p-value of <0.05 was considered statistically significant.

## RESULTS

Baseline demographic and clinical characteristics of the patient group, including primary diagnosis (traumatic brain injury, ischemic stroke, hemorrhagic stroke, tumor, or other), age, sex, and initial GCS, are summarized in Table 1.

**Table 1:** Baseline demographic and clinical characteristics of the patient.

Variable	Category	n (%)	Mean±SD	Median; Q1–Q3
Sex	Male	29 (58)	–	–
	Female	21 (42)	–	–
Age (years)	–	–	61.88±18.62	–
Initial GCS	–	–	9.32±4.43	8.5; 5–14
Subdural hematoma	Absent	43 (86)	–	–
	Present	7 (14)	–	–
Epidural hematoma	Absent	46 (92)	–	–
	Present	4 (8)	–	–
Subarachnoid hemorrhage	Absent	41 (82)	–	–
	Present	9 (18)	–	–
Ischemic stroke	Absent	36 (72)	–	–
	Present	14 (28)	–	–
Intracerebral hemorrhage	Absent	32 (64)	–	–
	Present	18 (36)	–	–
Intracranial mass	Absent	46 (92)	–	–
	Present	4 (8)	–	–
Hydrocephalus	Absent	50 (100)	–	–
	Present	0 (0)	–	–

Data are presented as n (%) for categorical variables and mean±SD or median; Q1–Q3 for continuous variables. GCS: Glasgow Coma Scale; SD: standard deviation; Q1–Q3: first and third quartiles.

The study comprised 50 patients with clinical and radiological suspicion of elevated ICP and 36 healthy volunteers. Significant differences in ONSD were observed between the patient and control groups for both eyes on all measured days (p<0.001). On average, the right ONSD measured 6.13±0.50 mm in patients and 4.23±0.22 mm in the control group, while the left ONSD measured 6.13±0.51 mm in patients and 4.23±0.21 mm in the control group (Table 2A).

**Table 2:** **(A)** Comparison of ONSD values according to study groups and **(B)** ONSD values compared with CT findings of patients on the 0th, 1st, 3rd, and 5th days of ICU admission.

A			
	Group C (n=36)	Group P (n=50)	p
	Mean±SD	Mean±SD	
ONSD Right	4.23±0.22	6.13±0.50	**<0.001** *
ONSD Left	4.23±0.21	6.13±0.51	**<0.001** *

Data represent the mean ONSD across all measured days,

* p<0.05 considered statistically significant. n: number of participants. SD: standard deviation; ONSD: optic nerve sheath diameter. The values indicated in bold represent statistically significant results (p<0.05).

**Table:** 

B					
CT finding	Day	ONSD right (mean±SD)	ONSD left (mean±SD)	p (right)	p (left)
Midline Shift	0	6.08±0.51 (No)/6.39±0.47 (Yes)	6.07±0.50 (No)/6.44±0.52 (Yes)	**0.049** *	**0.020** *
	1	6.05±0.48/6.38±0.51	6.03±0.49/6.42±0.53	**0.027** *	**0.013** *
	3	6.04±0.50/6.17±0.40	6.02±0.49/6.21±0.44	0.376	0.194
	5	6.04±0.46/6.28±0.53	6.01±0.45/6.27±0.46	0.111	0.062
Edema	0	5.86±0.47/6.36±0.44	5.92±0.50/6.34±0.49	**<0.001** *	**0.006** *
	1	5.76±0.40/6.33±0.45	5.84±0.48/6.31±0.50	**<0.001** *	**0.004** *
	3	5.66±0.31/6.23±0.43	5.68±0.37/6.22±0.43	**<0.001** *	**<0.001** *
	5	5.72±0.38/6.26±0.45	5.72±0.35/6.23±0.43	**<0.001** *	**<0.001** *

*ONSD: optic nerve sheath diameter*; CT: computed tomography; mean±SD: mean±standard deviation. *Values are presented as mean*±standard deviation (SD).

* p<0.05 is considered statistically significant. The values indicated in bold represent statistically significant results (p<0.05).

The correlation between ONSD and cranial CT findings was assessed on days 0, 1, 3, and 5. ONSD values in both eyes were significantly higher in patients with midline shift on days 0 and 1. In patients with cerebral edema, ONSD was significantly elevated on all assessed days (Table 2B).

In non-survivors, right-eye ONSD was significantly higher on day 5 (p=0.019), whereas left-eye ONSD was significantly elevated on days 3 and 5 (Table 3).

**Table 3:** Comparison of optic nerve sheath diameter and scoring systems according to mortality status.

Variable		Exitus (n=22) mean±SD	Survived (n=28) mean±SD	p
ONSD				
	Right Day 0	6.29±0.44	6.09±0.55	0.178
	Right Day 1	6.24±0.48	6.10±0.53	0.338
	Right Day 3	6.22±0.40	5.96±0.49	0.052
	Right Day 5	6.29±0.43	5.96±0.49	0.019*
	Left Day 0	6.32±0.44	6.07±0.57	0.106
	Left Day 1	6.29±0.45	6.09±0.58	0.279
	Left Day 3	6.25±0.42	5.93±0.47	0.018*
	Left Day 5	6.23±0.35	5.97±0.51	0.048*
GCS				
	Day 0	7.14±3.82	11.04±4.16	<0.001*
	Day 1	7.64±4.23	11.82±4.13	<0.001*
	Day 3	7.00±3.72	12.61±3.60	<0.001*
	Day 5	6.77±3.73	13.11±3.28	<0.001*
APACHE II				
	Day 0	20.41±6.08	10.75±5.91	<0.001*
	Day 1	19.73±5.12	9.68±5.33	<0.001*
	Day 3	21.05±6.04	8.43±4.80	<0.001*
	Day 5	21.77±7.45	7.64±4.54	0.001*
FOUR				
	Day 0	7.86±4.69	12.07±4.34	0.001*
	Day 1	8.36±5.09	12.75±4.30	<0.001*
	Day 3	7.73±4.67	13.64±3.61	<0.001*
	Day 5	7.32±4.95	14.18±3.22	<0.001*
SOFA				
	Day 0	7.00±2.09	3.50±2.32	<0.001*
	Day 1	6.95±2.42	2.64±1.95	<0.001*
	Day 3	7.41±2.68	2.50±1.97	<0.001*
	Day 5	7.36±2.87	2.21±1.85	<0.001*

*ONSD: optic nerve sheath diameter; GCS: Glasgow Coma Scale;* mean±SD: mean±standard deviation; n: number of participants; FOUR: Full Outline of UnResponsiveness; APACHE II: Acute Physiology and Chronic Health Evaluation II; SOFA: Sequential Organ Failure Assessment. Values are presented as mean±standard deviation (SD). Statistically significant differences (p<0.05) are indicated by asterisks based on the student's t-test. The values indicated in bold represent statistically significant results (p<0.05).

ICU scores over the measured days were compared according to survival status. GCS scores on days 0, 1, 3, and 5 were significantly higher in survivors. APACHE II scores decreased in survivors but increased in non-survivors. FOUR scores were higher and SOFA scores lower in survivors (Table 3).

On day 0 in the ICU, ONSD showed a negative correlation with GCS and FOUR scores. On day 1, right-eye ONSD was negatively correlated with GCS and FOUR, but positively correlated with APACHE II; left-eye ONSD was negatively correlated with GCS and FOUR. On day 3, right-eye ONSD correlated positively with APACHE II and SOFA and negatively with FOUR; left-eye ONSD correlated negatively with GCS and FOUR and positively with APACHE II and SOFA. On day 5, moderate correlations were observed between ONSD in both eyes and all scoring systems (Table 4). Correlation coefficients indicated weak-to-moderate associations that were statistically significant but of limited clinical relevance.

**Table 4:** Relationships between scoring systems and optic nerve sheath diameter measurements on intensive care unit admission days (0th, 1st, 3rd, and 5th days).

Day	Score	ONSD right (r)	ONSD left (r)	ONSD right (p)	ONSD left (p)
Day 0	GCS	-0.355	-0.350	**0.012** *	**0.013** *
	APACHE II	0.278	0.243	0.051	0.090
	FOUR	-0.379	-0.372	**0.007** *	**0.008** *
	SOFA	0.261	0.236	0.067	0.099
Day 1	GCS	-0.386	-0.395	**0.006** *	**0.005** *
	APACHE II	0.306	0.268	**0.031** *	0.060
	FOUR	-0.385	-0.395	**0.006** *	**0.005** *
	SOFA	0.258	0.271	0.071	0.057
Day 3	GCS	-0.264	-0.319	0.064	**0.024** *
	APACHE II	0.293	0.329	**0.039** *	**0.020** *
	FOUR	-0.292	-0.330	**0.039** *	**0.019** *
	SOFA	0.317	0.314	**0.025** *	**0.026** *
Day 5	GCS	-0.439	-0.398	**0.001** *	**0.004** *
	APACHE II	0.362	0.300	**0.010** *	**0.034** *
	FOUR	-0.428	-0.383	**0.002** *	**0.006** *
	SOFA	0.407	0.326	**0.003** *	**0.021** *

GCS: Glasgow Coma Scale; *ONCD: optic nerve sheath diameter;* r: correlation coefficient; FOUR: Full Outline of UnResponsiveness; APACHE II: Acute Physiology and Chronic Health Evaluation II; SOFA: Sequential Organ Failure Assessment;

* Statistical significance is denoted by an asterisk, Based on the Pearson correlation analysis (p<0.05). The values indicated in bold represent statistically significant results (p<0.05).

## DISCUSSION

In our study, ONSD measurements in ICU patients with clinical and radiological suspicion of elevated ICP, confirmed by cranial CT findings, were significantly higher than those of healthy controls. Patients with cerebral edema exhibited significantly elevated ONSD on all measured days, whereas those with midline shift showed elevations on days 0 and 1. ONSD was associated with mortality, and ICU scoring systems also correlated with outcomes in patients with suspected elevated intracranial pressure.

Ultrasonographic measurement of ONSD is a noninvasive method for evaluating elevated intracranial pressure. Previous studies have similarly reported significantly higher ONSD values in patients with suspected ICP elevation compared to healthy volunteers^8,9^.

Previous invasive ICP studies demonstrated that ONSD correlated with midline shift in patients with ICP >20 mmHg^10,11^. Although our study did not include invasive ICP measurements—a key limitation—we similarly observed elevated ONSD on days 0 and 1 in patients with midline shift. In follow-up CT scans, reduction in midline shift paralleled decreases in ONSD, suggesting that ONSD monitoring may aid in managing midline shifts. Previous research also indicated that ONSD serves as a marker of cerebral edema and treatment efficacy^12,13^; in our study, we likewise observed elevated ONSD in all patients with cerebral edema.

Increased ONSD has been associated with mortality. In patients with traumatic brain injury (TBI), each 1 mm increase in ONSD doubled hospital mortality, and similar associations have been reported in ischemic stroke, hemorrhagic stroke, and subarachnoid hemorrhage^14–16^. Consistent with these findings, our study observed a significant correlation between ONSD and mortality, particularly on ICU days 3 and 5. Therefore, elevated ONSD may indicate a higher risk of mortality in brain-injured ICU patients.

APACHE II is widely used to predict severity and mortality in ICU patients and has been shown to correlate with TBI outcomes, while SOFA scores are higher in fatal TBI and in deceased COVID-19 patients with brain injury^17–19^. The FOUR score has been reported to predict poor neurological outcomes more accurately than GCS^20,21^. In our study, APACHE II and SOFA scores were higher in non-survivor patients, consistent with previous findings. GCS and FOUR were also associated with mortality, although we could not assess their relative superiority. APACHE II, SOFA, GCS, and FOUR all appear useful for predicting poor outcomes. Importantly, we observed a consistent negative correlation between ONSD and FOUR score, a novel finding suggesting potential prognostic value.

Studies in patients with brain injury have demonstrated that ONSD correlates positively with ICP and negatively with GCS^22,23^. While some studies report correlations between ONSD and APACHE II or SOFA, others do not^22–24^. In our study, ONSD did not correlate with APACHE II on day 0, but showed positive correlations on days 3 and 5. This may reflect that APACHE II incorporates multiple parameters, unlike GCS, and that secondary brain injury evolves over time. Although SOFA is influenced by non-neurological systems, it may also increase as GCS decreases. Consequently, the ONSD–GCS correlation was expected and remained significant across all measured days. Importantly, ONSD consistently exhibited a strong negative correlation with FOUR scores throughout the study, highlighting its potential as a reliable prognostic marker. These findings suggest that the combined assessment of ONSD and FOUR could enhance early prognostication and guide clinical decision-making in brain-injured ICU patients more effectively than using either parameter alone.

## CONCLUSION

Ultrasonographic measurement of ONSD has emerged as a valuable, noninvasive, cost-effective, practical, and reliable and repeatable method. Bedside ONSD assessment in ICU settings is crucial for identifying patients with clinical and radiological suspicion of elevated intracranial pressure and for guiding timely treatment strategies, particularly in hemodynamically unstable patients. Nevertheless, the absence of invasive ICP monitoring as a gold standard limits definitive diagnostic accuracy. Future studies are warranted to validate optimal cutoff values and to delineate the precise role of ONSD in patient management. Importantly, the novel negative correlation between ONSD and FOUR score observed in our study may serve as an additional prognostic marker, potentially enhancing early risk stratification in brain-injured ICU patients.

### Limitations

This study has several limitations. First, invasive ICP monitoring, considered the gold standard, was not performed; thus, our findings could not be validated against direct ICP measurements, and sensitivity and specificity thresholds for ONSD could not be established. Second, the study was conducted at a single center with a relatively small sample size, potentially limiting the generalizability of the results. Finally, baseline patient diagnoses were heterogeneous (trauma, ischemic stroke, hemorrhage, and tumor), which may have influenced ONSD dynamics differently across subgroups. These limitations should be carefully considered when interpreting the findings.

## Data Availability

The datasets generated and/or analyzed during the current study are available from the corresponding author upon reasonable request.
